# The Scopoletin-HRP Fluorimetric Determination of H_2_O_2_ in Seawaters—A Plea for the Two-Stage Protocol

**DOI:** 10.3390/mps1010004

**Published:** 2017-12-01

**Authors:** Man Wu, George T. F. Wong, Yao-Chu Wu

**Affiliations:** 1 Key Laboratory of Global Change and Marine-Atmospheric Chemistry (GCMAC), Third Institute of Oceanography (TIO), State Oceanic Administration (SOA), Xiamen 361005, Fujian, China; 2 State Key Laboratory of Marine Environmental Science, Xiamen University, Xiamen 361005, China; 3 Research Center for Environmental Changes, Academia Sinica, Nankang, Taipei 115, Taiwan; ha119247@gmail.com; 4 Department of Ocean, Earth and Atmospheric Sciences, Old Dominion University, Norfolk, VA 23529, USA

**Keywords:** hydrogen peroxide, fluorimetric determination, scopoletin, horseradish peroxidase

## Abstract

A single solution protocol has been widely used for the fluorimetric determination of H_2_O_2_ in natural waters by its bleaching of the fluorescing scopoletin in the presence of the enzyme horseradish peroxidase (HRP). In this protocol, the reaction between scopoletin and H_2_O_2_ in the sample and the subsequent internal additions, and the measurements of the fluorescence are all carried out at a single pH in a fluorometer cell. It is found that this protocol is prone to four sources of possible error. The variability in the reaction stoichiometry between scopoletin and H_2_O_2_ in the presence of varying amounts of excess scopoletin, the effect of pH on the rate of reaction between scopoletin and H_2_O_2_, the photobleaching of scopoletin, and the de-activation of HRP. These possible sources of error can be circumvented in a two-stage protocol in which the reaction between H_2_O_2_ and scopoletin is carried out immediately upon sampling at a pH of 7, and the measurement of the fluorescence is carried out later on at a pH of 9. It should be the protocol of choice. Furthermore, in the two-stage protocol, after the initial reaction between H_2_O_2_ and scopoletin, the sample may be stored at room temperature for six days and at 4 °C for at least a month before its fluorescence is measured. This option can significantly reduce the logistics in the field.

## 1. Introduction

Hydrogen peroxide is found rather ubiquitously in seawater at concentrations ranging from undetectable (sub-nM) to 10^2^ nM [1,2,3,4,5,6,7]. A method that has been used extensively for its determination is the scopoletin–horseradish peroxidase (HRP) fluorimetric method [1,7,8,9,10,11,12,13,14]. In this method, a fluorescing agent, scopoletin, reacts with H_2_O_2_ in the presence of HRP to form a nonfluorescing compound [15]. The reduction in fluorescence is related to the concentration of H_2_O_2_, which can be quantified by internal additions. Holm et al. [8] described a single-solution protocol for the determination of H_2_O_2_ in groundwater samples in which the sample is analyzed immediately upon collection and the fluorometer cell is used as the reaction vessel. The reaction between H_2_O_2_ and scopoletin, including the internal additions, is carried out at a fixed pH. Subsequently, Zhang and Wong [1] reported a modified two-stage protocol in which the analytical scheme of Holm et al. [8] is separated into two stages. In the first stage, the reaction between scopoletin and H_2_O_2_ in the sample with and without an internal addition is carried out in the field in separate reaction vessels at the natural pH immediately upon sample collection. The sample may then be stored for up to four days. Then, in the second stage in the laboratory or in the field, the pH of the sample is adjusted to the optimal value of 9.2 and the fluorescence of the solution is measured. The purposes of this two-stage protocol are twofold: to make sample storage possible and to improve the sensitivity of the method. However, we have found that these two protocols frequently yield different results, especially when they are applied to seawater samples. Further examination indicates that there are other factors that favor the two-stage protocol. These factors include: the difference in the optimal pH for the reaction between scopoletin and H_2_O_2_ and for maximizing the fluorescence of scopoletin, the photobleaching of scopoletin in the fluorometer cell, the dependence of the reaction stoichiometry between H_2_O_2_ and scopoletin on the amount of excess scopoletin added, and the deactivation of the enzyme HRP during the analysis. Here, the effects of these processes are discussed and their implications on these two analytical protocols are assessed.

## 2. Materials and Methods

### 2.1. Apparatus

Two fluorometers with excitation light sources of different intensities were used for measuring the fluorescence of scopoletin. The Turner Model 10-AU-005-CE (Turner Designs, San Jose, CA, United States) is a filter fluorometer for both field and laboratory use. Its excitation light source is a low power 4W mercury lamp. The Hitachi Model F-7000 (Hitachi High-Technologies Corporation, Tokyo, Japan) is a high-performance research-grade scanning fluorescence spectrophotometer in which a high intensity 150 W xenon lamp is used as the excitation light source.

### 2.2. Reagents

All chemicals used are of ACS (American Chemical Society) reagent-grade. “H_2_O_2_-free” Milli-Q reagent grade water is prepared by the method of Zhang and Wong [16] and is used for the preparation of the reagents.

Standard 1 and 5 µM H_2_O_2_ solutions: a 1-ml portion of a 30% (*w*/*w*) solution of H_2_O_2_ was diluted to 1000 mL to make an approximately 0.01 M stock solution. This stock solution was standardized iodometrically by using iodate as the primary standard [17]. Standard 1 and 5 µM solutions were prepared from this standardized 0.01 M solution by serial dilutions. 

Scopoletin (10 and 100 μM): a 15 mg portion of scopoletin (Sigma, Saint Louis, MO, United States); 7-hydroxy-6-methoxy-2H-1-1benzopyran-2-one, C_10_H_8_O_4_, molecular weight (*M*_w_) = 192.2 g/mol) was dissolved in 500 mL of water to make a 150 μM stock solution. This stock solution was stored at 4 °C in the refrigerator until use. Ten and 100 μM solutions of scopoletin were prepared from the stock solution by serial dilutions immediately before use. These diluted solutions were stored at room temperature. 

Horseradish peroxidase (HRP) (600–1000 purpurogallin units (p.u.) mL^−1^): a 4 mg portion of HRP (Sigma, Type II, 150–250 p.u. mg^−1^) was dissolved in 1 mL of water. This solution was stored at 4 °C in a refrigerator until use. The catalytic effect of this reagent on the reaction between H_2_O_2_ and scopoletin could be maintained for at least two months. 

Saturated solution of Na_2_B_4_O_7_: a sufficient amount of Na_2_B_4_O_7_ was added to 100 mL of water so that a small amount of solid remained undissolved.

Phosphate buffer solution (pH 7.0): a 0.27 g portion of NaH_2_PO_4_·H_2_O and a 0.43 g portion of Na_2_HPO_4_ were dissolved in 100 mL of water to make a 0.05 M phosphate solution.

### 2.3. Procedures

#### 2.3.1. Single-Solution Protocol

The single-solution protocol for the determination of H_2_O_2_ follows the method of Holm et al. [8].

#### 2.3.2. Modified Single-Solution Protocol

Several minor modifications are applied to the method of Holm et al. [8]: the amount of scopoletin added is specified, an additional dose of HRP is added together with each internal addition of H_2_O_2_, and the reaction time between scopoletin and H_2_O_2_ is fixed at 3 min. Briefly, 5 mL of a sample, 0.05 mL of a 0.05 μM pH 7.0 phosphate buffer solution and 0.05 mL of a 100 μM solution of scopoletin are transferred to a fluorometer cell and the fluorescence at an excitation wavelength of 380 nm and an emission wavelength of 460 nm is measured in a Turner fluorometer (Turner Designs). Then, 0.01 mL of a 600–1000 p.u. mL^−1^ HRP solution is added to the cell and the fluorescence is read again after 3 min of reaction time. This is followed by three sequential additions of 0.1 mL of a 5 μM standard H_2_O_2_ solution and 0.01 mL of HRP solution to the cell and the fluorescence is measured 3 min after the addition of both solutions. The concentration of H_2_O_2_ in the sample is calculated as described by Holm et al. [8].

#### 2.3.3. Two-Stage Protocol

The two-stage protocol generally follows the method of Zhang and Wong [1]. A 5-mL aliquot of the sample and 0.5 mL of 10 µM scopoletin are transferred to each of three 15 mL light tight brown high density polyethylene reaction bottles. Then, 0.5 mL of water is added to two of the bottles while 0.5 mL of a 1 µM standard H_2_O_2_ solution is added to the third. Finally, 0.01 mL of 600–1000 p.u. mL^−1^ HRP solution is added to the last two bottles. These samples can be stored in the dark at room temperature for up to 6 days or refrigerated at 4 °C for up to 30 days prior to their analyses. When they are analyzed, 0.2 mL of a saturated Na_2_B_4_O_7_ buffer solution is added to each of the bottles to bring the pH of the solution to 9.2. Then, the fluorescence of each solution is read at an excitation wavelength of 380 nm and an emission wavelength of 460 nm. The concentration of H_2_O_2_ in the sample is calculated as described by Zhang and Wong [1].

## 3. Results and Discussion

### 3.1. The Stoichiometry in the Reaction between Hydrogen Peroxide and Scopoletin

**Figure 1:**
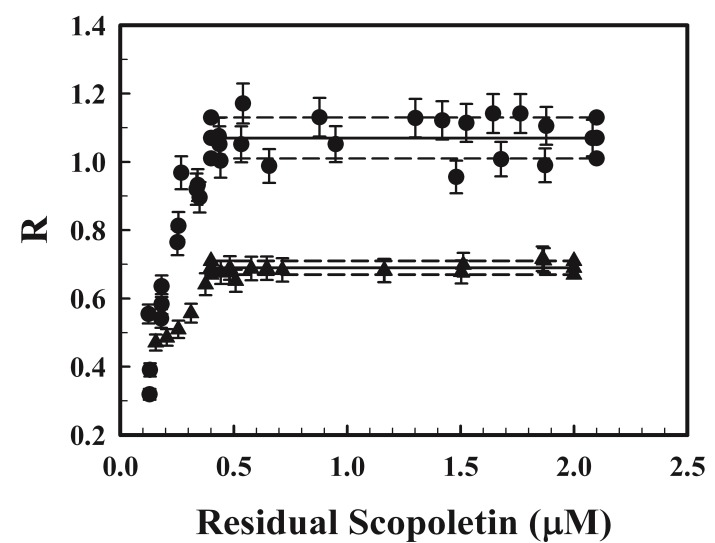
The effect of the concentration of residual scopoletin on the stoichiometry in the reaction between H_2_O_2_ and scopoletin in reagent-grade water (●) and in seawater (▲). R is the molar ratio of scopoletin lost to H_2_O_2_ reacted. Duplicate samples were at least analyzed at each level of residual scopoletin. —: average R at residual scopoletin exceeding 0.4 μM; - - -: one standard deviation from the mean.

The fluorescence of a 2.5 and a 0.6 µM scopoletin solution in water and natural seawater in the presence of 1.2–2 p.u. mL^−1^ of HRP and various amounts of H_2_O_2_ was measured. The amount of scopoletin that was destroyed by its reaction with H_2_O_2_ was estimated from the decrease in fluorescence. The mole ratio of the scopoletin lost to H_2_O_2_ reacted, and the amount of residual scopoletin left in the solution was calculated and the relationship between the two is shown in Figure 1. In both water and seawater, the mole ratio increased with increasing concentration of residual scopoletin and reached an approximately constant value of 1.07 ± 0.06 in water and 0.69 ± 0.02 in seawater when the residual scopoletin exceeded 0.4 µM. In both the single solution and the two-stage protocol for the determination of H_2_O_2_, a linear relationship between the decrease in fluorescence and the amount of H_2_O_2_ reacted is assumed. This assumption requires a constant mole ratio of scopoletin to H_2_O_2_ reacted, and thus an excess in scopoletin of at least 0.4 µM must be maintained. In the two-stage protocol, the amount of scopoletin added results in a 0.83 µM solution in the reaction mixture. The internally added H_2_O_2_ amounts to 0.08 µM. Thus, in order to maintain a constant molar ratio, the concentration of H_2_O_2_ in the sample must not exceed about 0.35 µM at a mole ratio of scopoletin to H_2_O_2_ reacted of 1 in water and 0.54 µM at a mole ratio of scopoletin to H_2_O_2_ reacted of 0.69 in seawater. This would cover the concentrations found in most seawater samples. The presence of an adequate amount of excess scopoletin can be safely assumed if, after each measurement, the fluorescence still exceeds 50% of the value without the addition of HRP. If samples with elevated concentrations of H_2_O_2_ are expected, the amount of scopoletin added needs to be adjusted upward accordingly. Nevertheless, there is an upper limit on the amount of excess scopoletin that should be added. Since the concentration of H_2_O_2_ in the sample is estimated from the decrease in fluorescence resulting from the reaction between H_2_O_2_ and scopoletin relative to the fluorescence of the added scopoletin, an excessively large amount of added scopoletin will lead to a small fractional decrease in fluorescence and a large uncertainty. 

In the single-solution protocol, the amount of scopoletin added was not clearly specified. It is given as “80% excess over the amount quenched by the natural H_2_O_2_” and the optimum volume of the internally added H_2_O_2_ quenched “approximately half of the fluorescence” [8]. If the concentration of H_2_O_2_ in the sample is 0.2 µM, a typical concentration in surface natural water, at a mole ratio between scopoletin and H_2_O_2_ reacted of 1, the amount of scopoletin added would be 180% × 0.2 or 0.36 µM and the residual scopoletin left after it that has reacted with the H_2_O_2_ in the sample would be 0.16 µM. This residual concentration is already below the threshold concentration of 0.4 µM, and the internally added H_2_O_2_ will further reduce it. Thus, in the single-solution protocol, the concentration of residual scopoletin is likely to be in the range where the molar ratio of scopoletin lost to H_2_O_2_ reacted is not at a constant value. Indeed, Holm et al. [1] reported variable molar ratios as low as 0.1. If a decreasing molar ratio, as a result of the decreasing amount of residual scopoletin at the different steps in the protocol, does occur, it is a potentially significant source of error and may lead to overestimations and degraded precision in the measurement. It is interesting to note that the mole ratio of scopoletin lost to H_2_O_2_ reacted was close to 1 in pure water when a sufficient amount of excess scopoletin was present as previously reported [15]. However, in seawater, the ratio only reached 0.7, suggesting that there may also be a matrix effect on the stoichiometric ratio of the reaction.

### 3.2. The Effect of pH on the Reaction between Hydrogen Peroxide and Scopoletin

**Figure 2:**
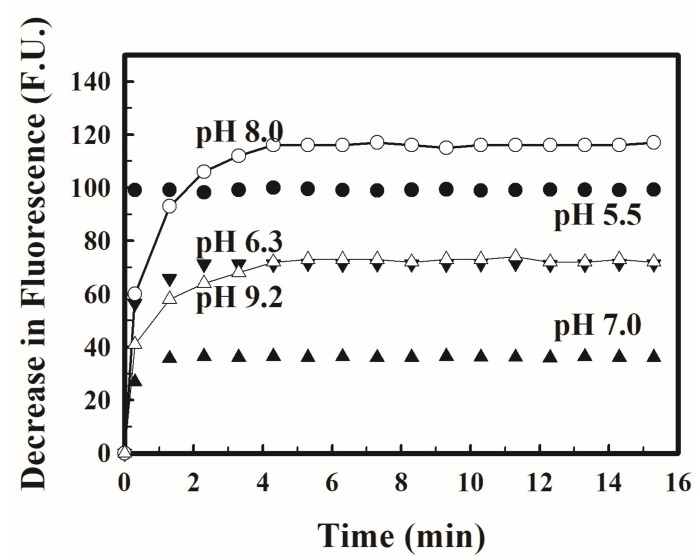
The time course of the decrease in the fluorescence, in fluorescence unit (f.u.) of the reaction mixture of H_2_O_2_ and scopoletin at different pH. pH was adjusted by the addition of HCl and/or NaOH. (●: pH 5.5; ▲: pH 6.3; ▼: pH 7.0; ○: pH 8.0; △: pH 9.2). Fluorescence at the different pH were not recorded contemporaneously using the same setting and thus could not be compared with each other.

**Figure 3:**
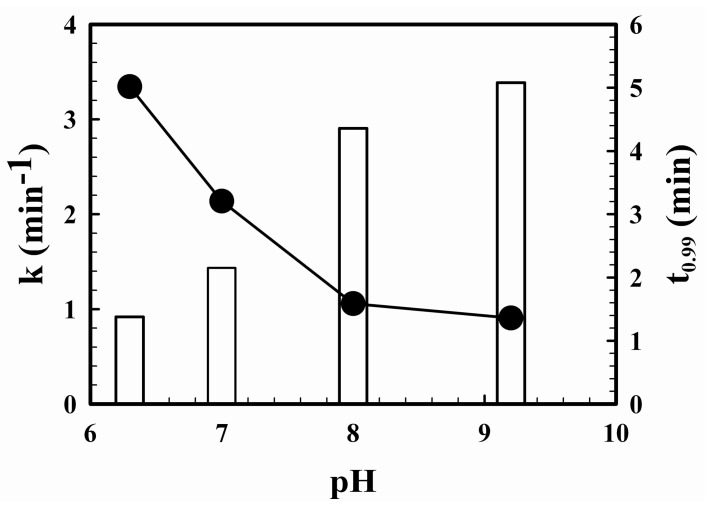
The effect of pH on the rate of the reaction between hydrogen peroxide and scopoletin. ●: first order rate constant (left axis); bars: time needed for the reaction to reach 99% completion. k: first order rate constant; t_0.99_: time needed for the reaction to reach 99% completion.

The pH of a solution containing 0.1 µM of H_2_O_2_ and 2 µM of scopoletin was adjusted to pH 5.5, 7, 8 and 9.2 by the addition of appropriate amounts of a 0.1 M NaOH and/or 0.1 M HCl solution. The natural pH of the H_2_O_2_-scopoletin solution without addition of any acid or alkali was 6.3. Then, 0.01 mL of a 600–1000 p.u. mL^−1^ HRP solution was added to each of these mixtures. The fluorescence of these solutions at the optimal excitation and emission wavelength at each of these pH values was then followed with time and the resulting time courses of reduction in fluorescence are shown in Figure 2. In all cases, the decrease in fluorescence increased with time and eventually reached a constant maximum value. At pH 5.5, the maximum reduction in fluorescence, indicating the completion of the reaction, was reached approximately instantaneously. At the other pH values, the changes in fluorescence could be approximated as a first order reaction. The resulting first order rate constants decreased with increasing pH (Figure 3). The corresponding reaction times for the reaction to reach 99% of completion were 1.4, 2.1, 4.4 and 5.1 min at pH 6.3, 7.0, 8.0 and 9.2. Thus, the reaction time decreased with decreasing pH. The reaction time was shortened at a lower pH, but the sensitivity of the method was increased at a higher pH [1], so a minimum reaction time and a maximum sensitivity cannot be achieved simultaneously when a single pH is used in the analysis as in the single-solution protocol. In the two-stage protocol, this difficulty is circumvented as the completion of the reaction is assured, since it is allowed to proceed at the low natural pH of scopoletin for up to days while the sensitivity of the analysis is maximized since the fluorescence is measured after the pH of the reaction mixture has been adjusted to the optimal value. For this reason alone, the two-stage protocol should be the protocol of choice. However, another potentially more significant issue in the single-solution protocol [8] is the possibility of incomplete reactions. At the pH of 7.0 used in the single-solution protocol [8], a reaction time in excess of 2 min is needed to ensure completion of the reaction. However, the reaction time for the reaction between scopoletin and the H_2_O_2_ in the sample and the H_2_O_2_ in each standard internal addition has not been clearly specified [8,12]. If it is not stringently controlled, a variable and unpredictable error may appear.

### 3.3. Photobleaching of Scopoletin

**Figure 4:**
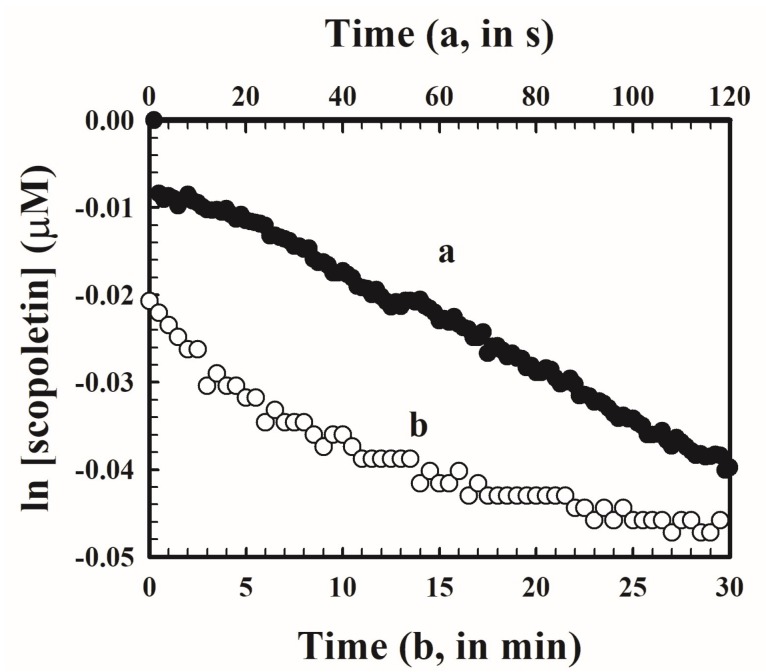
The time course of change in the natural logarithm of scopoletin concentration (in μM) by photobleaching in two fluorometers. (a) ●: Hitachi Model F-7000 fluoresence spectrophotometer, upper scale (in seconds); (b) ○: Turner Model 10-AU-005-CE filter fluorometer, lower scale (in minutes)

The decomposition of scopoletin by photobleaching was studied by following the time courses of change in fluorescence of scopoletin solutions, at initial concentrations ranging between 0.5 and 2 µM, at the excitation and emission wavelengths for the determination of H_2_O_2_ in a fluorometer cell that is exposed to the light source in a Turner Model 10-AU filter fluorometer or a Hitachi Model F-7000 fluorescence spectrophotometer (Hitachi High-Technologies Corporation). The fluorescence intensity was converted to the corresponding concentrations of scopoletin and the results at one initial concentration of scopoletin are shown in Figure 4. The observed changes in concentration with time may be represented by first order kinetics well. The resulting first order reaction rate constants are listed in Table 1. In the Hitachi fluorescence spectrophotometer where a high intensity 150 W xenon lamp was used as the light source, there was a conspicuous decrease in concentration with time. The first order rate constant was 3 × 10^−4^ s^−1^ at all the initial concentrations. In the Turner Model 10-AU filter fluorometer, a low-power 4 W mercury lamp was used as the light source and the first order rate constant was only 1.3 × 10^−5^ s^−1^, or about thirty times smaller than that in the Hitachi fluorescence spectrophotometer. In the single-solution protocol, the exposure of the reaction mixture to the light source is prolonged. Any photobleaching of scopoletin while the fluorescence of the sample is measured will appear as an apparent additional amount of H_2_O_2_ and lead to an overestimation in the concentration of H_2_O_2_ in the sample. On the other hand, photobleaching of scopoletin that occurs during the internal additions of H_2_O_2_ will increase the specific fluorescence and lead to an underestimation. The net result is uncertain and likely will be variable. In fluorimetric analysis, a light source with the highest light intensity is usually preferred, as it will provide the highest sensitivity. However, in this case, if the single-solution protocol is used, a fluorometer with a weaker light source should be preferred in order to minimize the effect of photobleaching. At the slow rate of photobleaching, the effect should be negligible in the Turner fluorometer at an exposure time of several minutes. In the two-stage protocol, the exposure time of the reaction mixture to the light source is sufficiently short such that the effect of photo-bleaching will be minimal regardless of which spectrophotometer is used, and the higher sensitivity provided by the more intense light source may be utilized.

### 3.4. Deactivation of Horseradish Peroxidase

The stability of the enzymatic activity of HRP for catalyzing the reaction between scopoletin and H_2_O_2_ during the prolonged exposure of the reaction mixture to heat and light in the Turner fluorometer in the single-solution protocol was examined by estimating the specific fluorescence in samples of reagent grade water and seawater with and without the addition of another dose of HRP upon the internal additions of H_2_O_2_. The results are shown in Table 2. Invariably, the specific fluorescence was higher when HRP was added together with each internal addition of H_2_O_2_ before the fluorescence was read. Furthermore, when additional HRP was added, the specific fluorescence was similar in the same type of water, at 369 ± 8 fluorescence units (f.u.) µM^−1^ in reagent grade water and 587 ± 17 f.u. µM^−1^ in seawater, regardless of the concentration of H_2_O_2_ in the sample. Without adding another dose of HRP, the specific fluorescence was lower and more variable, at 348 ± 18 f.u. µM^−1^ in reagent grade water and 372 ± 77 f.u. µM^−1^ in seawater. These behaviors are consistent with the deactivation of the HRP during the analysis so that the HRP added initially can no longer effectively and reproducibly catalyze the reaction between scopoletin and the H_2_O_2_ added in the internal additions in the later stage of the analysis. The deactivation of HRP has been noted by the supplier of the chemical [18] and its effect on the fluorimetric determination of H_2_O_2_ in the stored sample has been noticed previously [1]. However, its effect on the single solution protocol has not been evaluated. On the other hand, the deactivation of HRP is irrelevant to the two-stage protocol since HRP is used to catalyze the reaction only once when it is added to the sample immediately after sample collection.

### 3.5. Sample Storage

**Figure 5:**
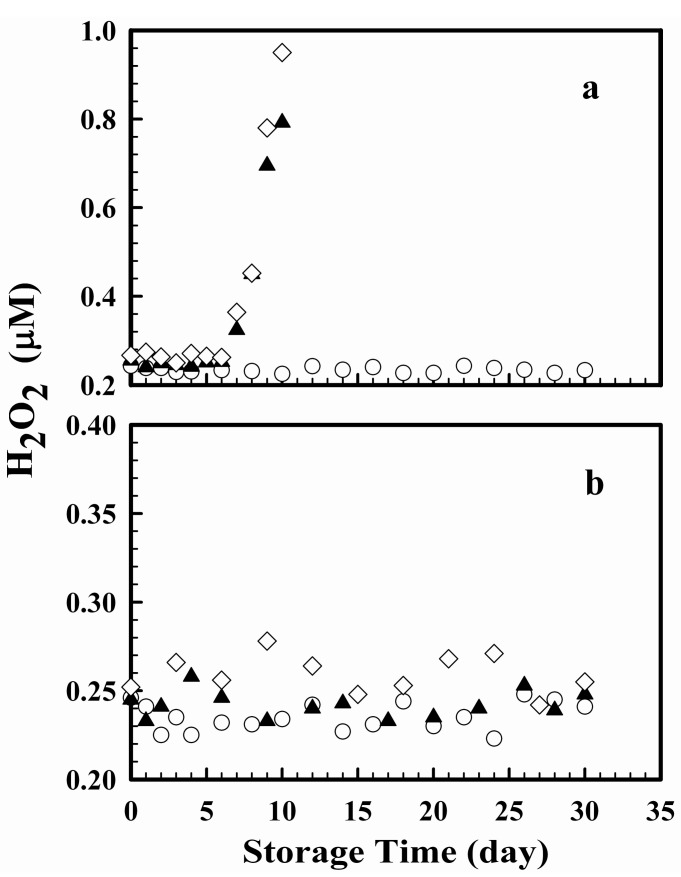
Variations in the concentration of H_2_O_2_ in reagent grade water (○), seawater (▲) and estuarine water (◇) at (**a**) 24 °C and (**b**) 4 °C upon storage.

Known amounts of H_2_O_2_ were added to samples of “H_2_O_2_-free” reagent grade water, aged estuarine water and aged surface seawater collected from the South China Sea. Scopoletin and HRP were then added to subsamples of these samples as specified in the two-stage protocol. One of the samples was analyzed immediately for the concentration of H_2_O_2_. The other subsamples were stored either at room temperature (24 °C) or in a refrigerator at 4 °C. The concentrations of H_2_O_2_ in these subsamples were quantified after various times of storage. The results are shown in Figure 5. In a previous study [1], the concentration upon storage at room temperature was followed to a maximum storage time of four days and no significant change in the concentration was observed. In this study, the maximum storage time at room temperature was extended to 10 days. In the first six days, the results of natural water samples were similar to those reported previously as the concentration of H_2_O_2_ stayed constant at an average concentration of 0.248 ± 0.006 µM in the seawater sample and 0.265 ± 0.006 µM in the estuarine water sample. The standard deviations were within the analytical uncertainty of ±5%. However, at storage times longer than six days, the concentration of H_2_O_2_ increased rapidly with time, probably as a result of the decomposition of the scopoletin. The concentration after 10 days of storage was three times those in the first six days of storage. Thus, sample storage should be limited to less than six days at room temperature. In contrast, in Milli-Q reagent-grade water, the concentration of H_2_O_2_ stayed constant at 0.234 ± 0.006 μM for at least 30 days. At a storage temperature of 4 °C, there was no significant change in the concentration of H_2_O_2_ up to the maximum storage time of 30 days used in the experiment. The average concentrations of H_2_O_2_ over those 30 days of storage time were 0.242 ± 0.008 µM in the seawater sample, 0.259 ± 0.011 µM in the estuarine water sample, and 0.235 ± 0.008 µM in the Milli-Q reagent-grade water. The variations were within the analytical uncertainty of the method and the concentrations found were indistinguishable from those measured within the first six days of storage at room temperature. Thus, in the two-stage protocol, under refrigeration, the storage time of the natural water samples may be extended to at least a month.

### 3.6. Comparison of Results Obtained from the Single-Solution Protocols and the Two-Stage Protocol

The concentrations of H_2_O_2_ in samples of reagent grade water and aged surface seawater from the South China Sea with and without the addition of a known amount of H_2_O_2_ were determined with the two-stage protocol. The samples with added H_2_O_2_ were also analyzed by using both single-solution protocols, and the results are shown in Table 3. The two-stage protocol yielded highly precise results. The average deviation from the mean was about ±0.003 μM at a concentration level of around 0.1 μM in duplicate samples and a quantitative recovery of the added H_2_O_2_, averaging 102 ± 4%, was found. When the modified single-solution protocol was used, comparable results, averaging about 104% of the concentrations by the two-stage protocol, were found in reagent-grade water. However, in seawater, noticeably higher concentrations, averaging about 126% of those by the two-stage method, were found. Even higher concentrations were found when the unmodified single-solution method was used. The deviations were larger in seawater than in reagent-grade water. These results indicate that significant overestimations in the concentration of H_2_O_2_ may occur when the single-solution protocol is used and the discrepancies may be larger in the analyses of seawater. Even with the added precautions, the modified single solution protocol cannot eliminate the overestimations totally in seawater. The two-stage protocol should be the protocol of choice. It is free from the potential sources of error discussed previously and it allows the samples to be stored for an extended period of time after relatively simple pretreatments.

## 4. Conclusions

When the scopoletion-HRP fluorimetric method is used to determine H_2_O_2_ in seawater, the traditional single solution protocol described by Holm et al. [8] is prone to four sources of possible errors. First, as the amount of scopoletin decreases during the internal additions of H_2_O_2_, the stoichiometry of scopoletin to H_2_O_2_ reacted does not stay constant if the amount of excess scopoletin drops below 0.4 µM. Secondly, while the fluorescence of scopoletin is higher at a more basic pH, the rate of the reaction between scopoletin and H_2_O_2_ is slower. In the single solution protocol where a single neutral to basic pH is used, incomplete reaction may occur if the reaction time is not carefully controlled. Thirdly, photobleaching of scopoletin may occur during the prolonged exposure of the reaction mixture to the light source while it is kept in the fluorescence spectrophotometer. The lost scopoletin may appear as an apparent H_2_O_2_ in the sample. This effect is especially acute in high performance research grade fluorescence spectrophotometers fitted with high intensity light sources. Fourthly, HRP in the reaction mixture may undergo deactivation as the reaction mixture is kept at or above room temperature in the fluorescence spectrophotometer. The modified two-stage protocol reported here can not only circumvent these possible sources of possible error in the single solution protocol, but also extend the storage time of samples for the determination of H_2_O_2_ to up to at least a month. At concentrations of around 0.02 and 0.1 μM, the precision of the modified method were about ±15% and ±3% at concentrations of 0.02 and 0.1 μM, respectively. The virtually quantitative recovery of added H_2_O_2_ indicates that the method is highly accurate. 

## Figures and Tables

**Table 1:** The first order reaction rate constants in the photobleaching of scopoletin in the two fluorometers.

Fluorometer	C_0_ (µM )	k (10^−4^ s^−1^)	r
Hitachi Model F7000	2.003	3	0.9987
	1.487	3	0.9895
	0.994	3	0.9960
	0.493	3	0.9835
			
Turner Model10-AU	0.980	0.13	0.9375
	0.494	0.13	0.9465

C_0_: initial concentration of scopoletin; k: the first order rate constant; r: the correlation coefficient of the natural logarithm of concentration against time.

**Table 2:** Effect of the addition of horseradish peroxidase (HRP) upon the internal additions of H_2_O_2_ on the specific fluorescence of scopoletin.

Sample	Specific Fluorescence (f.u. μM^−1^)	
	Without Additional HRP	With Additional HRP
RW	357, 369	370, 379
RW + 0.1 μM H_2_O_2_	334, 333	367, 361
Average	348 ± 18	369 ± 8
SW	438, 438	582, 609
SW + 0.1 μM H_2_O_2_	306, 305	568, 590
Average	372 ± 77	587 ± 17

RW: reagent-grade water; SW: surface seawater; Specific fluorescence: decrease in fluorescence per µM of internally added H_2_O_2_.

**Table 3:** Determination of H_2_O_2_ in reagent grade water and seawater by three protocols.

Sample	H_2_O_2_ Added μM	Protocol A Found μM	Protocol B Found μM	B/A %	Protocol C Found μM	C/A %
RW	0	0.013, 0.011				
Average		0.012 ± 0.001				
	0.10	0.116, 0.115	0.120, 0.121		0.158, 0.152	
Average		0.116 ± 0.001	0.120 ± 0.001	103	0.155 ± 0.005	134
Recovery		0.104 ± 0.002				
% Recovery		104 ± 2				
RW	0	0.021, 0.020				
Average		0.021 ± 0.001				
	0.10	0.120, 0.118	0.128, 0.122		0.156, 0.162	
Average		0.119 ± 0.001	0.125 ± 0.004	105	0.159 ± 0.005	134
Recovery		0.098 ± 0.002				
% Recovery		98 ± 2				
SW	0	0.024, 0.021				
Average		0.023 ± 0.002				
	0.10	0.130, 0.124	0.158, 0.161		0.291, 0.271	
Average		0.127 ± 0.003	0.159 ± 0.002	125	0.281 ± 0.014	221
Recovery		0.104 ± 0.005				
% Recovery		104 ± 5				
SW	0	0.022, 0.016				
Average		0.019 ± 0.004				
	0.10	0.121, 0.127	0.146, 0.154		0.285, 0.290	
Average		0.124 ± 0.004	0.150 ± 0.005	121	0.288 ± 0.004	232
Recovery		0.105 ± 0.008				
% Recovery		105 ± 8				
SW	0	0.015, 0.012				
Average		0.014 ± 0.002				
	0.10	0.114, 0.117	0.140, 0.150		0.219, 0.252	
Average		0.111 ± 0.005	0.145 ± 0.007	130	0.235 ± 0.023	212
Recovery		0.097 ± 0.007				
% Recovery		97 ± 7				

RW: reagent-grade water; SW: surface seawater; protocol A: two-stage protocol; protocol B: modified single-solution protocol; protocol C: single-solution protocol; B/A: Average concentration by protocol B/Average concentration by protocol A; C/A: Average concentration by protocol C/Average concentration by protocol A.
